# Recursive Partitioning Analysis of Fractional Low-Frequency Fluctuations in Narcolepsy With Cataplexy

**DOI:** 10.3389/fneur.2018.00936

**Published:** 2018-11-02

**Authors:** Xiao Fulong, Lu Chao, Zhao Dianjiang, Zou Qihong, Zhang Wei, Zhang Jun, Han Fang

**Affiliations:** ^1^Department of Respiratory and Critical Care Medicine, Sleep Medicine Center, Peking University People's Hospital, Beijing, China; ^2^Department of Radiology, Peking University International Hospital, Beijing, China; ^3^Center for MRI Research, Academy for Advanced Interdisciplinary Studies, Peking University, Beijing, China; ^4^PKU-Upenn Sleep Center, Peking University International Hospital, Beijing, China; ^5^Department of Neurology, Peking University People's Hospital, Beijing, China

**Keywords:** narcolepsy, functional magnetic resonance imaging, fractional low-frequency fluctuations, recursive partitioning analysis, receiver operating characteristic curve analysis

## Abstract

**Objective:** To identify narcolepsy related regional brain activity alterations compared with matched healthy controls. To determine whether these changes can be used to distinguish narcolepsy from healthy controls by recursive partitioning analysis (RPA) and receiver operating characteristic (ROC) curve analysis.

**Method:** Fifty-one narcolepsy with cataplexy patients (26 adults and 25 juveniles) and sixty matched heathy controls (30 adults and 30 juveniles) were recruited. All subjects underwent a resting-state functional magnetic resonance imaging scan. Fractional low-frequency fluctuations (fALFF) was used to investigate narcolepsy induced regional brain activity alterations among adult and juveniles, respectively. Recursive partitioning analysis and Receiver operating curve analysis was used to seek the ability of fALFF values within brain regions in distinguishing narcolepsy from healthy controls.

**Results:** Compared with healthy controls, both adult and juvenile narcolepsy had lower fALFF values in bilateral medial superior frontal gyrus, bilateral inferior parietal lobule and supra-marginal gyrus. Compared with healthy controls, both adult and juvenile narcolepsy had higher fALFF values in bilateral sensorimotor cortex and middle temporal gyrus. Also juvenile narcolepsy had higher fALFF in right putamen and right thalamus compared with healthy controls. Based on RPA and ROC curve analysis, in adult participants, fALFF differences in right medial superior frontal gyrus can discriminate narcolepsy from healthy controls with high degree of sensitivity (100%) and specificity (88.9%). In juvenile participants, fALFF differences in left superior frontal gyrus can discriminate narcolepsy from healthy controls with moderate degree of sensitivity (57.1%) and specificity (88.9%).

**Conclusion:** Compared with healthy controls, both the adult and juvenile narcolepsy showed overlap brain regions in fALFF differences after case-control comparison. Furthermore, we propose that fALFF value can be a helpful imaging biomarker in distinguishing narcolepsy from healthy controls among both adults and juveniles.

## Introduction

Narcolepsy is a chronic sleep disorder, characterized by excessive daytime sleepiness, cataplexy, sleep paralysis, hypnagogic, and hypnopompic hallucination and disturbed nocturnal sleep. A deficient endogenous orexin system due to neuronal degeneration in the hypothalamus is the main pathophysiology of the narcolepsy in the human (1). It is indicated that loss of hypocretin is thought to be an underlying cause to the sleep-related changes and cataplexy, also deficiency in hypocretin system can result in the abnormal cognition and emotion observed in narcolepsy patients (2).

In the past decades, neuroimaging techniques have played an important role in the understanding of physiology and pathology in human sleep medicine (3, 4). Changes in brain structure and function have been investigated in hypersomnia and narcolepsy (5–8). These studies include the measurement of brain structure, such as voxel-based morphometry, diffusion tensor imaging, and metabolic studies using spectroscopy, as well as functional view, such as positron emission tomography (PET), single photon emission computed tomography (SPECT), and functional magnetic resonance imaging (fMRI). Detection of local dysfunction is crucial to the clinical research and clinical practice. Results from previous neuroimaging studies suggested that reduction of hypocretin can lead to attenuation in both resting state glucose metabolism and perfusion within cortex (9). Abnormal perfusion and glucose metabolism in the hypothalamus and prefrontal cortex has been detected among narcolepsy using PET and SPECT (5, 10). A very recent PET research in a large group of junior narcolepsy patients observed that abnormality in many frontal and subcortical brain areas, exhibited significantly correlation with neuro-cognition performance (7).

Resting state fMRI can provide information about spontaneous brain activity by assessment of blood oxygen level dependent (BOLD) signal fluctuations. The resting BOLD signal fluctuations are thought to represent spontaneous and functional process, although on a slower time response. Brain regions involved in specific task or stimuli display coherent low BOLD signal fluctuations in the resting state. Amplitude of low-frequency fluctuations (ALFF), in which the square root of power spectrum was integrated in a low-frequency range, was developed for detecting the local intensity of BOLD signal fluctuations (11). ALFF has already been applied to fMRI studies about attention deficit hyperactivity disorder and Alzheimer's disease, also in the exploration of neural mechanism of sleep disorders, such as insomnia, sleep deprivation and sleep apnea (11–14). Although ALFF was considered to be a useful tool in detecting the regional neural activity, physiological noise, such as the repetition times in MRI scan and so on, are not critically considered in the ALFF calculation. Therefore, a modified calculation called fractional amplitude of low-frequency fluctuation (fALFF), which means the ratio of the power spectrum of low frequency (0.01–0.08 Hz) to that of the entire frequency range, has been proven to suppress non-specific noise components and improve the effectiveness in exploring local BOLD signals (15). Considering the robustness and stability of ALFF and fALFF calculation, both the ALFF and fALFF can be indicated as potential biomarkers in neuroimaging studies (16).

Recursive partitioning analysis (RPA) could provide a simple, straightforward and intuitive method to classify subjects or to identify synergistic interaction among numerous factors (17, 18). RPA is considered to be a machine learning method and usually requires a large sample to establish a classification model from a training data and verify this model by another test sample. RPA can be realized through computer and many medical care studies have used RPA to detect prognostic and risk factors (19, 20), as well as diagnosis in imaging study (21). Classification and regression tree (CRT) analysis is a kind of tree-building technique from RPA to the generation of clinical decision rules (22). It is a non-parametric method for multi-model numerical data and categorical predictors, also suitable for managing the interactions between predictors which are crucial in determining the outcome. The CRT is a relatively data-driven machine learning calculation, which produces decision tree model easy to interpret (22).

In the present study, we hypothesized that fALFF has the ability to indicate narcolepsy induced neurobiological mechanism with the location of altered neural brain activity, and further distinguish narcolepsy from healthy controls with excellent sensitivity and specificity. Specifically, classification and regression tree form recursive portioning analysis (RPA) and receiver operating characteristic (ROC) curve analysis were used to investigate and validate the ability of fALFF values in distinguishing narcolepsy from healthy controls.

## Materials and methods

### Participants

Twenty six adult narcolepsy patients and another 25 juvenile patients were recruited as newly diagnosed narcolepsy with cataplexy according to the International Classification of Sleep Disorders (ICSD)-3 (23) from the Sleep Medicine Center of the Respiratory Department at Peking University People's Hospital between November 2016 and February 2018. Another 60 gender- and age- matched healthy volunteers (30 juveniles and 30 adults) were recruited from the hospital and community (Table 1). None of healthy controls had any consistent psychiatric or neurologic condition producing excessive daytime sleepiness. All narcolepsy cases were the first-time visitors and previously had never taken psychiatric stimulant medications. The clinical diagnosis of narcolepsy was made by a sleep specialist based on both excessive daytime sleepiness lasting more than 3 months and defined history of cataplexy, according to the International Classification of Sleep Disorders criteria for narcolepsy. The final diagnosis of narcolepsy was confirmed by a polysomnogram followed by a next day multiple sleep latency test (MSLT). Detailed information, including the presentation of excessive daytime sleepiness and cataplexy, hypnagogic hallucinations, and sleep paralysis, were obtained from patients.

**Table 1:** Demography for narcolepsy patients and healthy controls.

	**Demography**	**Narcolepsy patients**	**Healthy controls**	***P*-value**
**Adult**	Gender (female/male)	8/18	12/18	0.58
	Age (year)	25.77 ± 6.64	25.37 ± 4.31	0.786
	Education (year)	10.35 ± 2.3	10.95 ± 3.1	0.488
	Duration of EDS (year)	7 (2.3,12)	-	-
	Duration of cataplexy (year)	6.3 (2.2,10.3)	-	-
**Juvenile**	Gender (female/male)	5/20	6/24	0.63
	Age (year)	14 ± 2.7	13.3 ± 2.3	0.459
	Education (year)	8 ± 2.7	7.6 ± 2.7	0.688
	Duration of EDS (year)	5.4 (2.9,6.8)	-	-
	Duration of cataplexy (year)	3.8 (1.7, 6.8)	-	-

*The P value for gender distribution in the two groups was obtained by the Chi-square test. The P values for differences in age and years of education in the two groups were obtained by the two-sample t test. Values are expressed as the mean±SD or median (25%quartile, 75%quartile). EDS, excessive daytime sleepiness*.

The exclusion criteria for both narcolepsy and normal subjects were as follows: (1) other sleep disorders, such as obstructive sleep apnea, insomnia; (2) diabetes, and chronic obstructive pulmonary disease and heart disease; (3) neurological diseases and structural lesion based on brain MRI findings; (4) psychosis disorder; (5) alcohol, drug, and substance abuse; (6) inborn or congenital diseases; (7) MRI contraindications, such as claustrophobia or foreign implants in the body.

This research was performed in accordance with the ethical guidelines of the Declaration of Helsinki (version 2002) and was approved by the Medical Ethics Committee of Peking University People's University. All participants provided written informed consent.

### Imaging data acquisition

MRI examination was performed exactly following the daytime MSLT. MRI data were obtained on 3T (3 Tesla) scanner (Siemens, Skyra, Germany) using an 8-channel brain phased-array coil. Foam pads were used to minimize subject head motion, and headphones were used to reduce scanner noise. Resting BOLD MRI scans were obtained with gradient-echo planar imaging (TR = 2030 ms, TE = 30ms, slice = 33, FA = 90°, FOV = 224 × 224 mm, matrix = 64 × 64, voxel size = 3.5 × 3.5 × 3.5), after the BOLD MRI scan, a high-resolution T1-weighted structural image was acquired with the following parameters: TR = 1900 ms, TE = 2.55 ms, FA = 9°, FOV = 240 × 240 mm, thickness = 1 mm. A total 240 brain functional volumes were acquired in the resting BOLD MRI scans. All participants, including patients and controls were asked to resist sleeping in order to remain fully awake (5, 24), not to move and keep eye open during the whole MRI scan, supervised clinically and by video both a physician and a technician during the whole process. In addition, we controlled for the absence of emotional triggering factors during the entire process to avoid cataplexy-related events.

### Functional imaging data analysis

Functional MRI data preprocessing was performed using the Data Processing & Analysis for Resting State Brain Imaging V2.1 [DPABI V2.1 (25)], which works with Statistical Parametric Mapping (SPM8) implemented in the MATLAB (The Math Works, Inc., Natick, MA, USA) platform. The first 5 functional volume images of each subject's dataset were discarded, then the remaining fMRI data were corrected for slice timing and realigned for motion correction. Participants with head motion exceeding 3 mm in translation and 3° in rotation were rejected. Anatomical and functional images were manually reoriented to the anterior commissure, and structural images were co-registered to the functional images for each subject using a linear transformation. Also the transformed structural images were segmented into gray matter, white matter, and cerebrospinal fluid by the new segmentation in SPM8. For adult participants, the functional images were normalized to the standard Montreal Neurological Institute (MNI) space template with a resampling voxel size of 3 × 3 × 3 mm. For juvenile participants, the functional images were normalized to the CCHMC pediatric brain template (irc.cchmc.org, The imaging research center, Cincinnati Children's Hospital Medical Center) (26) with a resampling voxel size of 3 × 3 × 3 mm. The normalized functional images were smoothed using a Gaussian filter 4 mm FWHM. Linear trends were removed within each time series. The covariates were regressed out from the time series of every voxel, including the white matter signal, cerebrospinal fluid signal, Friston 24 motion parameters (27, 28) and the global signal. The calculation of the fALFF have been reported in previous studies (15). After fALFF calculation, the time series were filtered using typical temporal bandpass (0.01–0.1Hz) to reduce low-frequency drift, physiological high-frequency respiratory and cardiac noise. To reduce the global effects of variability across the participants, the individual fALFF map was transformed to Z score (minus the global mean value and then divided by the standard deviation) other than simply being divided by the global mean (15).

## Statistical analysis

### Demographic data

The demographic data differences between narcolepsy and healthy controls were computed by independent two sample *t*-test with the IBM Statistical Package for the Social Sciences 23.0 software (IBM SPSS Inc., Chicago, IL, USA). We set the significance level at *P* < 0.05. Values are expressed as the *mean* ± *SD* or *median (25%quartile, 75%quartile)*.

### Between group differences in FALFF

A two-sample *t*-test was performed between narcolepsy and controls using age, gender, and years of education as nuisance covariates to assess case-control comparison in fALFF among adults and juveniles, respectively, corrected for false discovery rate (FDR, *P* < 0.05).

### Recursive partitioning analysis (RPA)

Narcolepsy cases and healthy controls were randomly split into testing data and validation data in the proportion of 7:3, respectively. Testing data was used to develop decision tree model by recursive partitioning analysis (70%) and validation data was used to test the developed model (30%). In the analysis of between group fALFF differences, the brain regions showing statistically significant in adults or juveniles were selected as ROI seeds, respectively, then the mean fALFF value in the region of interesting (ROI) regions were extracted. Recursive partitioning analysis was performed using mean fALFF values within ROI regions showing group differences in adults or juveniles, respectively. We chose the Classification and Regression Trees (CRT) technique in the process of RPA to define narcolepsy or control. The criteria for splitting node including the following: child nodes derived from a parent node should be as homogeneous as possible with the dependent variables, corresponding cut-off points should result in the minimal *P* value, provides the minimal *P* value was ≤ 0.0001 (29). Terminal nodes were identified to a class when the significant level of comparison between 2 terminal nodes was >0.05 (29). As for the validation data, sensitivity, specificity, false positive rate (FPR), false negative rate (FNR), positive predictive value (PPV), negative predictive value (NPV), and accuracy were calculated according to the fALFF value cut-off obtained on the basis of developed decision tree model. ROC analysis was applied to measure the discrimination of the decision tree model. RPA process and ROC curve statistical analysis was performed with R (http://www.R-project.org) and Empower-Stats software (www.empowerstats.com, X&Y solutions, Inc., Boston, MA, USA).

## Results

### Demographic data

As shown in Table 1, there were no significant differences between narcolepsy and healthy controls in age, gender, years of education.

### Differences in FALFF between narcolepsy and healthy controls

**Figure 1:**
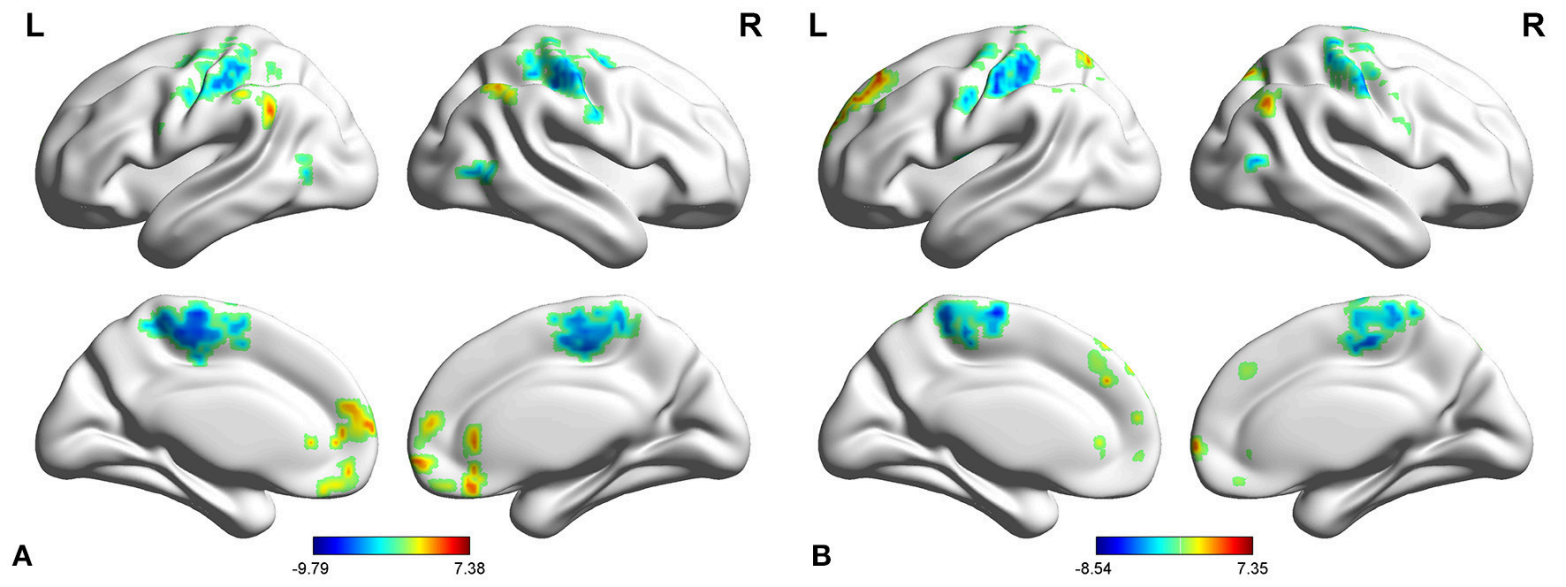
Two-sample *t*-test results for fALFF between narcolepsy and healthy controls among adults **(A)** and juveniles **(B)**.

**Figure 2:**
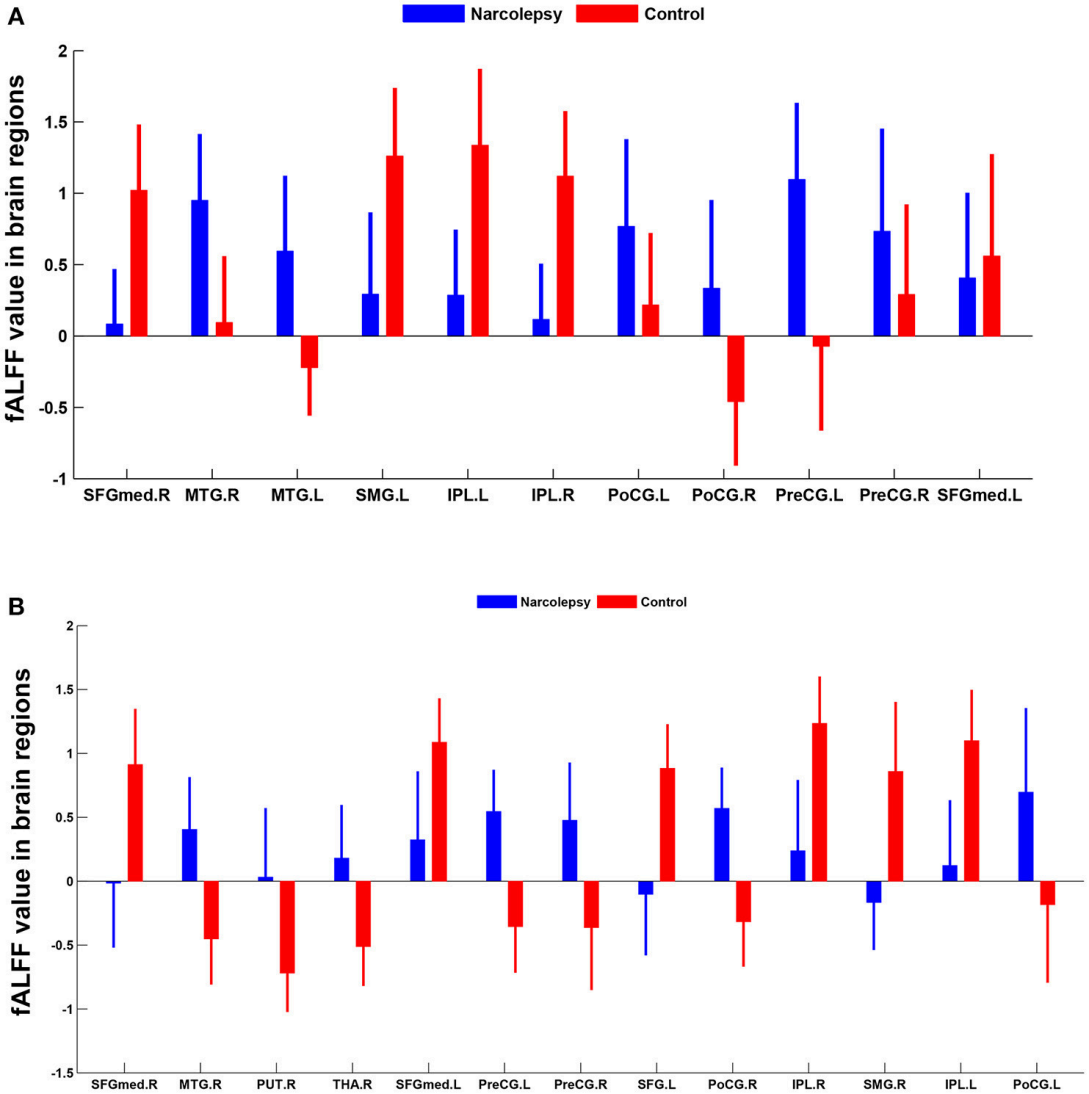
Mean fALFF values differences in regional brain areas between narcolepsy and healthy controls among adults **(A)** and juveniles **(B)**. SFGmed, medial superior frontal gyrus; MTG, middle temporal gyrus; SMG, supra-marginal gyrus; IPL, inferior parietal lobule; PoCG, postcentral gyrus; PreCG, precentral gyrus; PUT, putamen; THA, thalamus; SFG, superior frontal gyrus. L, left; R, right.

In adult participants, compared with healthy controls, narcolepsy had lower fALFF values in bilateral medial superior frontal gyrus (SFGmed), bilateral inferior parietal lobule (IPL) and left supra-marginal gyrus (SMG). Compared with healthy controls, narcolepsy had higher fALFF values in bilateral sensorimotor cortex (SMC) and bilateral middle temporal gyrus (MTG) (Figures 1A, 2A and Table S1). In juvenile participants, compared with healthy controls, narcolepsy had lower fALFF values in bilateral medial superior frontal gyrus, bilateral inferior parietal lobule, left superior frontal gyrus (SFG), and right supra-marginal gyrus. Compared with healthy controls, narcolepsy had higher fALFF values in bilateral sensorimotor cortex, right middle temporal gyrus, right putamen and right thalamus (Figures 1B, 2B and Table S1).

### Recursive partitioning analysis (RPA) of FALFF values

**Figure 3:**
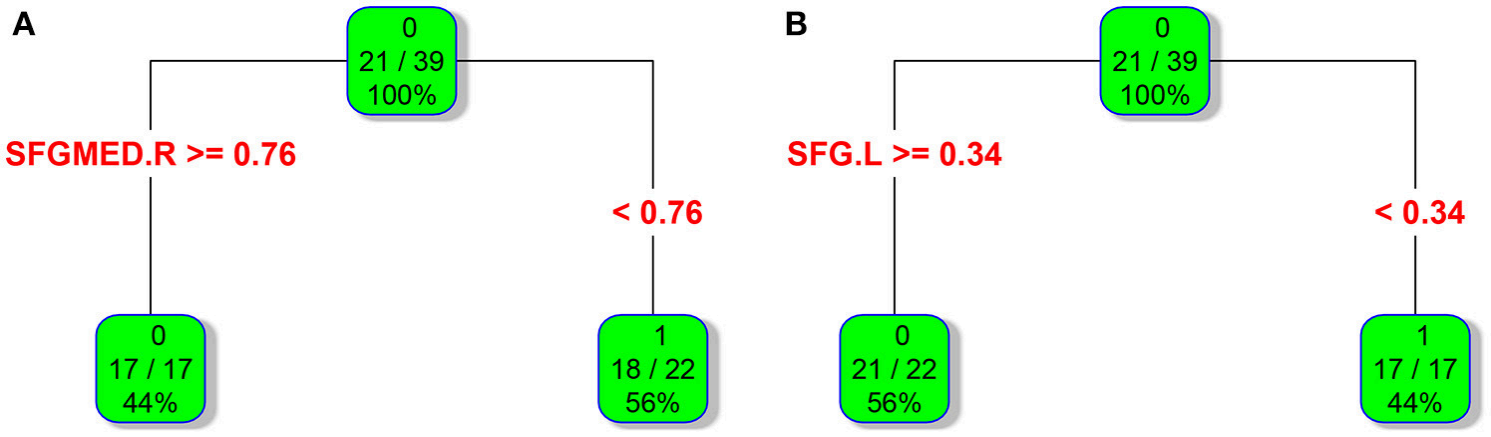
RPA process results of classification and regression tree about fALFF values within two brain regions between narcolepsy and healthy controls among adults **(A)** and juveniles **(B)**. As for each box, the binary value in the top represents group (0, healthy controls; 1, narcolepsy); as for the ratio in the middle, numerator means the size of patients or controls in the box and it is reflected by the top binary value, denominator means sample size in the box; the percentage in the bottom means the percentage of each box sample size in the tree. As for a brain region (or a splitting node), the cut-off fALFF value was also shown. SFGmed, medial superior frontal gyrus; SFG, superior frontal gyrus. L, left; R, right.

**Figure 4:**
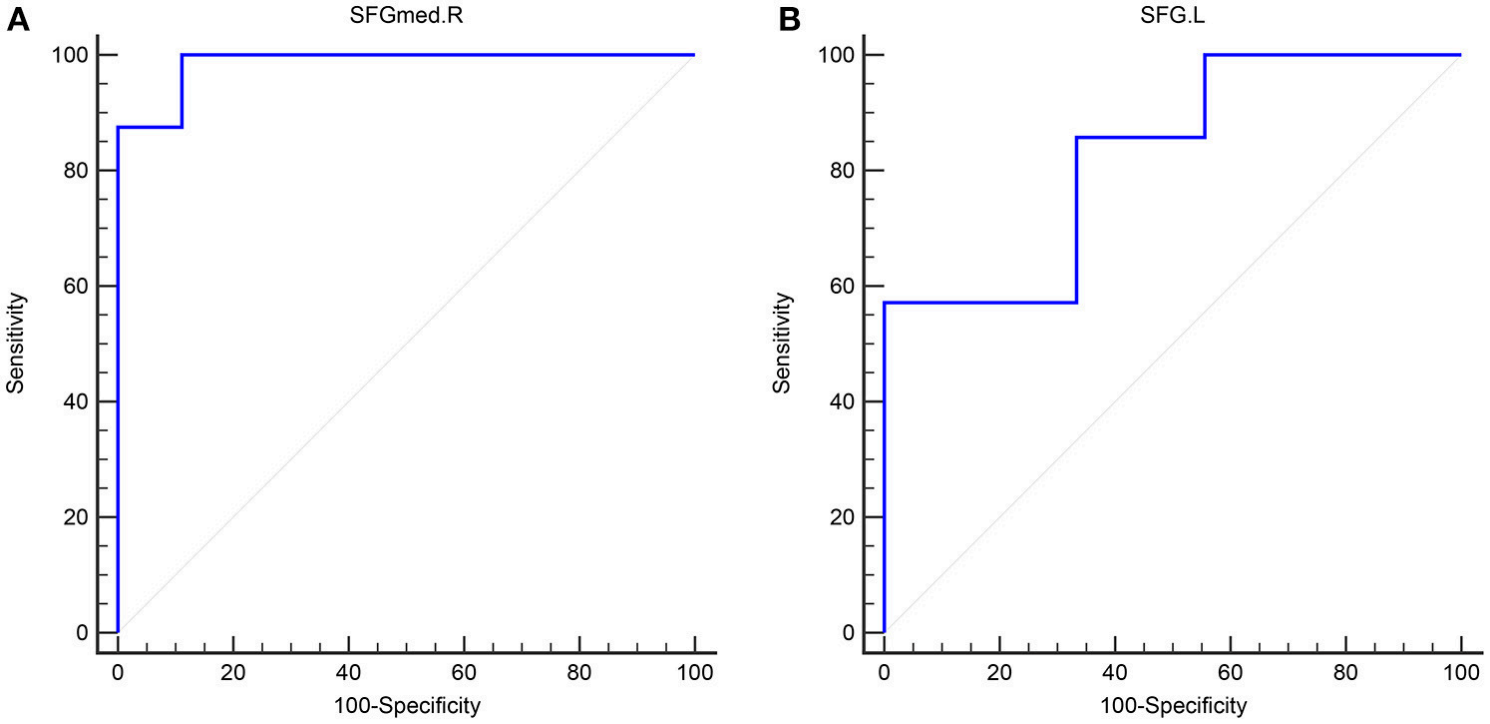
The validation ROC curve of each brain region based on results from RPA analysis for distinguishing narcolepsy from healthy controls among adults **(A)** and juveniles **(B)**. SFGmed, medial superior frontal gyrus; SFG, superior frontal gyrus. L, left; R, right.

In adult participants, 18 narcolepsy cases (18/26, 69%) and 21 healthy controls (21/30, 70%) were used as testing data in the recursive partitioning analysis and the developed decision tree model was shown in Figure 3A. In juvenile participants, 18 narcolepsy cases (18/25, 72%) and 21 healthy controls (21/30, 70%) were used as testing data in the recursive partitioning analysis and the developed decision tree model was shown in Figure 3B. The cut-off fALFF values of these nodes were also shown in the decision tree model (Figure 3). In adult participants, 8 narcolepsy cases (8/26, 31%) and 9 healthy controls (9/30, 30%) were used as validation data in the ROC analysis of developed decision tree model (Figure 4A). When decision tree model applied to the validation data, it revealed the sensitivity was 100%, and the specificity 88.9%. The FPR was 11.1% and the FNR was 0. Meanwhile the model showed the PPV of 88.9%, the NPV of 100%, and the accuracy of 94.1% (Table 2). In juvenile participants, 7 narcolepsy cases (7/25, 28%) and 9 healthy controls (9/30, 30%) were used as validation data in the ROC analysis of developed decision tree model (Figure 4B). When decision tree model applied to the validation data, it revealed the sensitivity was 57.1%, and the specificity 88.9%. The FPR was 11.1% and the FNR was 42.9%. Meanwhile the model showed the PPV of 80%, the NPV of 72.7% and the accuracy of 75% (Table 2).

**Table 2:** Validation for Decision tree model about fALFF differences in brain regions between narcolepsy and healthy control.

**Group**	**Brain regions**	**Sn %**	**Sp %**	**FPR %**	**FNR %**	**PPV %**	**NPV %**	**Accuracy %**	**AUC**
Adult	SFGmed.R	100	88.9	11.1	0	88.9	100	94.1	0.986
Juvenile	SFG.L	57.1	88.9	11.1	42.9	80	72.7	75	0.825

*Sn, sensitivity; Sp, specificity; FPR, false positive rate; FNR, false negative rate; PPV, positive predictive value; NPV, negative predictive value; AUC, area under the curve; SFGmed.R, right medial superior frontal gyrus; SFG.L, left superior frontal gyrus*.

## Discussion

This study compared fALFF differences in both adult and juvenile narcolepsy patients with those in a group of matched healthy controls. Specially, compared with healthy controls, we identified some overlap brain regions showing significantly different fALFF values in both adult and juvenile narcolepsy patients, including bilateral medial superior frontal gyrus, bilateral sensorimotor cortex, supra-marginal gyrus, middle temporal gyrus, and bilateral inferior parietal lobule. It has been revealed that utility of ROC curve analysis in neuroimaging can distinguish one group of participants from another group of participants (13, 14). Furthermore, by using recursive partitioning analysis and ROC curve analysis, we speculated that the fALFF values in some brain regions were excellent in discriminating narcolepsy subjects from healthy controls in both adults and juvenile with high AUC value.

Low-frequency fluctuation measures are widely used for the assessment of group differences in many previous resting-state studies focusing on clinical case-control comparison (16). Furthermore, standardization has been identified effective in eliminating the dependency of fALFF values on subjective motion (16), so Z score of fALFF (i.e., standardization of fALFF) was used in the between group comparison. In both the adult and juvenile participants, narcolepsy patients showed decreased fALFF in bilateral SFGmed, bilateral supra-marginal gyrus and bilateral IPL compared with healthy controls, while narcolepsy patients showed increased fALFF in bilateral SMC and bilateral middle temporal gyrus compared with healthy controls. Both the medial frontal cortex, supra-marginal gyrus, and parietal lobe are abundant in hypocretin projection (30, 31), which can explain the reduced fALFF value in these regions among narcolepsy due to hypocretin deficiency, consistent with two previous positron emission tomography studies (7, 8). Increased fALFF in bilateral SMC, extending to bilateral paracentral lobule (Figure 1), may be a compensation of hypocretin deficiency in motor cortex among narcolepsy, although a contradictory result has been reported hypo-activity in sensorimotor cortex in narcolepsy by transcranial magnetic stimulation (TMS) in a previous study (32). Increased glucose metabolism in temporal lobe has been indicated in previous studies (7, 24), which was consistent with increased fALFF value in middle temporal gyrus in the present study result. Such increased fALFF value or hyper-metabolism in temporal lobe may be related to transient activation of this region, compensation for the hypocretin deficiency.

Meanwhile, especially in juvenile narcolepsy, higher fALFF value in right putamen and right thalamus can be detected compared with healthy controls. Putamen is a component of the salience network (24). The salience network is responsible for integration of sensory and attention information, initiation of responses to significant stimuli as a function of top-down attention and cognitive control process (33, 34). The salience network is also thought to maintain the tonic of alertness, correlated with sympathetic regions (35, 36). Also thalamus is a core brain region responsible for sympathetic regulation, arousal, and wakefulness (35, 36). In our resting-state fMRI study, for drug-free narcolepsy patients, it requires a specific order to resist sleepiness during the MRI scan. The increased fALFF in putamen and thalamus among juvenile narcoleptic patients reinforced its major role in the reservation of the awaking status and the activated sympathetic nervous system. The relatively increased fALFF may reflect the patients' subjective effort to maintain vigilance, consistent with already reported in obstructive sleep apnea (37), in Kleine-Levin syndrome (38) and in PET narcolepsy study (24).

Interestingly, based on the results of classification and regression tree from recursive partitioning analysis, validated ROC curve analysis indicates that in adult participants the fALFF value in right SFGmed alone could discriminate narcolepsy from healthy controls with high degree of sensitivity, specificity, and accuracy (Figure 4A). Also in juvenile participants, the validated ROC curve indicated that the fALFF value in left SFG alone could also discriminate narcolepsy from healthy controls with moderate degree of sensitivity, specificity and accuracy (Figure 4B). Although there were many brain regions showing fALFF value differences between groups, just one brain region was necessary to discriminate narcolepsy from healthy controls in adults and juveniles, respectively.

The present study has some limitations. Small sample size and single setting should be the first consideration in limitations, especially in the validation data, small sample size may lead to some bias and confounding. Also participants in this study all come from China, which may potentially be not applicable to other ethnic groups. While being fully awake during the whole examination as controlled clinically and by video, but the vigilance state was not monitored through synchronous EEG recording during the MRI scan. Our design cannot directly confirm the absence of short fluctuations in alertness and even short sleep events during the MRI process. Further simultaneous EEG-fMRI studies based on large samples are necessary to confirm our preliminary results on fALFF value differences between narcolepsy and healthy controls, also to compare narcolepsy patients with other hypersomnia and sleep deprivation in resting wakefulness.

To conclude, compared with healthy controls, both the adult and juvenile narcolepsy showed overlap brain regions in fALFF differences after case-control comparison. Furthermore, we propose that fALFF value can be a helpful imaging biomarker in distinguishing narcolepsy from healthy controls among both adults and juveniles.

## Ethics statement

This study was approved by the Ethical Committee of the Peking University People's Hospital.

## Author contributions

ZJ and HF designed the study. XF, LC, ZD, ZQ, and ZW carried out the study. XF performed data analysis and wrote the manuscript.

### Conflict of interest statement

The authors declare that the research was conducted in the absence of any commercial or financial relationships that could be construed as a potential conflict of interest.

## Supplementary material

Table S1 Significant differences in fALFF value between narcolepsy patients and healthy controls.

Table S2 Original data for testing RPA and validated ROC analysis.

## Abbreviations

fMRI: Functional magnetic resonance imaging
PET: Positron emission tomography
SPECT: Single photon emission computed tomography
BOLD: Blood oxygen level dependent
ROC: Receiver operating characteristic
MSLT: Multiple sleep latency test
RPA: Recursive partitioning analysis
AUC: Area under the curve
