# Supplementary material for: Recursive Partitioning Analysis of Fractional Low-Frequency Fluctuations in Narcolepsy With Cataplexy
Source: Front Neurol. 2018 Nov 2;9:936. doi: 10.3389/fneur.2018.00936 (PMC6225790; doi:10.3389/fneur.2018.00936)
Supplement: Table S1 — Significant differences in fALFF value between narcolepsy patients and healthy controls. [file Table_1.DOC]

**Table S1. Significant differences in fALFF value between narcolepsy patients and healthy controls**

|  | **Condition** | **L/R** | **Brain regions** | **MNI coordinates** | | | **Size(voxels)** | **T value** |
| --- | --- | --- | --- | --- | --- | --- | --- | --- |
|  |  |  |  | **X** | **Y** | **Z** |  |  |
| **Adult** | HC>Narcolepsy | R | Medial superior frontal cortex | 3 | 54 | 12 | 28 | 7.38 |
|  | HC<Narcolepsy | R | Middle temporal gyrus | 42 | -60 | -3 | 16 | -5.22 |
|  | HC<Narcolepsy | L | Middle temporal gyrus | -42 | -57 | 0 | 16 | -5.55 |
|  | HC<Narcolepsy | L | Postcentral gyrus | -30 | -21 | 45 | 236 | -9.79 |
|  | HC<Narcolepsy | R | Postcentral gyrus | 24 | -33 | 51 | 234 | -4.37 |
|  | HC<Narcolepsy | L | Precentral gyrus | -36 | -18 | 54 | 122 | -6.94 |
|  | HC>Narcolepsy | L | Supra-marginal gyrus | -57 | -51 | 30 | 12 | 6.34 |
|  | HC>Narcolepsy | R | Inferior parietal lobule | 48 | -57 | 51 | 56 | 6.62 |
|  | HC>Narcolepsy | L | Inferior parietal lobule | -48 | -51 | 54 | 44 | 5.99 |
|  | HC<Narcolepsy | R | Precentral gyrus | 45 | -21 | 51 | 82 | -8.53 |
|  | HC>Narcolepsy | L | Medial superior frontal cortex | -5 | 49 | 13 | 74 | 7.38 |
| **Juvenile** | HC>Narcolepsy | R | Medial superior frontal cortex | 0 | 42 | -3 | 21 | 6.12 |
|  | HC<Narcolepsy | R | Middle temporal gyrus | 39 | -51 | 3 | 21 | -5.45 |
|  | HC<Narcolepsy | L | Postcentral gyrus | -54 | -21 | 45 | 220 | -8.54 |
|  | HC<Narcolepsy | R | Postcentral gyrus | 33 | -30 | 51 | 158 | -7.93 |
|  | HC<Narcolepsy | L | Precentral gyrus | -33 | -18 | 54 | 86 | -8.54 |
|  | HC>Narcolepsy | R | Inferior parietal lobule | 27 | -72 | 54 | 31 | 6.99 |
|  | HC>Narcolepsy | L | Inferior parietal lobule | -30 | -66 | 57 | 29 | 6 |
|  | HC<Narcolepsy | R | Precentral gyrus | 42 | -6 | 36 | 17 | -5.28 |
|  | HC>Narcolepsy | L | Medial superior frontal cortex | 0 | 42 | 18 | 14 | 4.75 |
|  | HC<Narcolepsy | R | Putamen | 24 | 21 | -3 | 29 | -5 |
|  | HC<Narcolepsy | R | Thalamus | 18 | -12 | 9 | 29 | -5.52 |
|  | HC>Narcolepsy | L | Superior frontal gyrus | -18 | 48 | 39 | 61 | 6.76 |
|  | HC>Narcolepsy | R | Supra-marginal gyrus | 63 | -24 | 48 | 20 | 7.35 |

T value, statistical value of peak voxel (*P*<0.05, False discovery rate corrected). HC, healthy control
